# Supplementary material for: End Sequence Analysis Toolkit (ESAT) expands the extractable information from single-cell RNA-seq data
Source: Genome Res. 2016 Oct;26(10):1397–410. doi: 10.1101/gr.207902.116 (PMC5052061; doi:10.1101/gr.207902.116)
Supplement: Supplemental Material [file supp_gr.207902.116_Supp_Materials.tar.gz › ESAT-esat_v0.1/doc/html/functions_vars_d.html]

ESAT: Class Members - Variables


ESAT


- Main Page
- Related Pages
- Packages
- Classes
- Files

- Class List
- Class Index
- Class Hierarchy
- Class Members

- All
- Functions
- Variables
- Enumerator

- a
- b
- c
- d
- e
- f
- g
- h
- i
- k
- l
- m
- n
- o
- p
- q
- r
- s
- t
- u
- v
- w


All Classes Namespaces Files Functions Variables Enumerator Pages

### - d -

- data
  : broad.pda.seq.clip.SampleData
  , broad.pda.seq.protection.SampleData
- DEFAULT\_ALPHABET\_SIZE
  : broad.core.motif.PositionWeightColumn
- DEFAULT\_GAP\_EXTEND\_PENALTY
  : umms.core.alignment.FeatureSequenceAlignment
  , umms.core.alignment.SmithWatermanAlignment
- DEFAULT\_GAP\_OPEN\_PENALTY
  : umms.core.alignment.FeatureSequenceAlignment
  , umms.core.alignment.SmithWatermanAlignment
- DEFAULT\_INS\_SIZE\_PVAL
  : broad.pda.seq.segmentation.ContinuousDataAlignmentModel
- DEFAULT\_INSERT\_SIZE\_FUDGE
  : broad.pda.seq.segmentation.ContinuousDataAlignmentModel
- DEFAULT\_MAKE\_GENE\_OVERLAP
  : broad.pda.seq.segmentation.ContinuousDataAlignmentModel
- DEFAULT\_MATCH\_SCORE
  : umms.core.alignment.FeatureSequenceAlignment
  , umms.core.alignment.SmithWatermanAlignment
- DEFAULT\_MIN\_MAPPING\_QUALITY
  : broad.pda.seq.segmentation.ContinuousDataAlignmentModel
- DEFAULT\_MISMATCH\_SCORE
  : umms.core.alignment.FeatureSequenceAlignment
  , umms.core.alignment.SmithWatermanAlignment
- DEFAULT\_REGION\_TOTAL
  : umms.core.model.score.CountScore
  , umms.core.model.score.PoissonEnrichmentScore
- DEFAULT\_VALUE
  : umms.core.model.score.AbstractListScore
- degenerateCodonStarts
  : broad.core.multiplealignment.AlignmentExtractor
- DEPLETED
  : broad.pda.seq.rap.RatioPermutationPeakCaller
  , broad.pda.seq.rap.SkellamKLPeakCaller
- DIAGONAL
  : jaligner.Directions
- DISCRETE\_EXTENSION
  : broad.pda.seq.rap.CollectAnnotationEnrichments


---

Generated on Fri Aug 15 2014 15:11:17 for ESAT by  

 1.8.7
